# Building cooperative learning to address alcohol and other drug abuse in Mpumalanga, South Africa: a participatory action research process

**DOI:** 10.1080/16549716.2020.1726722

**Published:** 2020-03-02

**Authors:** Oladapo Oladeinde, Denny Mabetha, Rhian Twine, Jennifer Hove, Maria Van Der Merwe, Peter Byass, Sophie Witter, Kathleen Kahn, Lucia D’Ambruoso

**Affiliations:** a Aberdeen Centre for Health Data Science (ACHDS) Institute of Applied Health Sciences, School of Medicine, Medical Sciences and Nutrition, University of Aberdeen, Aberdeen, Scotland, UK; b MRC/Wits Rural Public Health and Health Transitions Research Unit (Agincourt), School of Public Health, Faculty of Health Sciences, University of the Witwatersrand, Johannesburg, South Africa; c Maria Van Der Merwe Consultancy Group, South Africa; d Department of Epidemiology and Global Health, Umeå University, Umeå, Sweden; e Institute for Global Health and Development, Queen Margaret University, Edinburgh, Scotland, UK

**Keywords:** South Africa, alcohol and drug abuse, community participation, health systems, rural, primary health care

## Abstract

**Background**: Alcohol and other drug (AOD) abuse is a major public health challenge disproportionately affecting marginalised communities. Involving communities in the development of responses can contribute to acceptable solutions.

**Objectives**: To: (1) document forms, processes, and contexts of engaging communities to nominate health concerns and generate new knowledge for action; (2) further build participation in the local health system by reflecting on and adapting the process.

**Methods**: PAR was progressed with 48 community stakeholders across three rural villages in the MRC/Wits Agincourt Health and Socio Demographic Surveillance System (HDSS) in Mpumalanga, South Africa. A series of workshops explored community-nominated topics, systematised lived experience into shared accounts and considered actions to address problems identified. Photovoice was also used to generate visual evidence. Narrative and visual data were thematically analysed, situated within practice frameworks, and learning and adaption elicited.

**Results**: AOD abuse was identified as a topic of high priority. It was understood as an entrenched social problem with destructive effects. Biopsychosocial impacts were mapped and related to unemployment, poverty, stress, peer pressure, criminal activity, corruption, and a proliferating number of taverns. Integrated action agendas were developed focussed on demand, supply, and harm reduction underpinned by shared responsibility among community, state, and non-state actors. Community stakeholders appreciated systematising and sharing knowledge, taking active roles, developing new skills in planning and public speaking, and progressing shared accountability processes. Expectations required sensitive management, however.

**Conclusion**: There is significant willingness and capacity among community stakeholders to work in partnership with authorities to address priority health concerns. As a process, participation can help to raise and frame issues, which may help to better inform action and encourage shared responsibility. Broader understandings of participation require reference to, and ultimately transfer of power towards, those most directly affected, developing community voice as continuous processes within social and political environments.

## Background

This paper reports on community-based research in rural South Africa on alcohol and other drug (AOD) abuse. AOD abuse is a significant global health challenge. The UN estimates that quarter of a billion adults used drugs at least once in 2015, with over 200,000 drug-related deaths reported in 2014 [1]. Risks include addiction, overdose, interpersonal violence, risky sexual behaviours, injuries, accidents, hepatitis, TB, and HIV/AIDS [1]. Alcohol use and abuse is a co-occurring problem with similar patterns of addiction and harms. Alcohol differs in important ways related to its legality, with a concentrated industry that disregards a public health approach. Alcohol-related diseases and injuries take approximately 3.3 million lives every year, and it is a causal factor in more than 200 diseases and conditions [2]. In sub-Saharan Africa, alcohol abuse accounts for 6.4% of all deaths and 4.7% of all Disability Adjusted Life Years (DALYs), which is expected to rise in the future [3].

**Figure 1.**
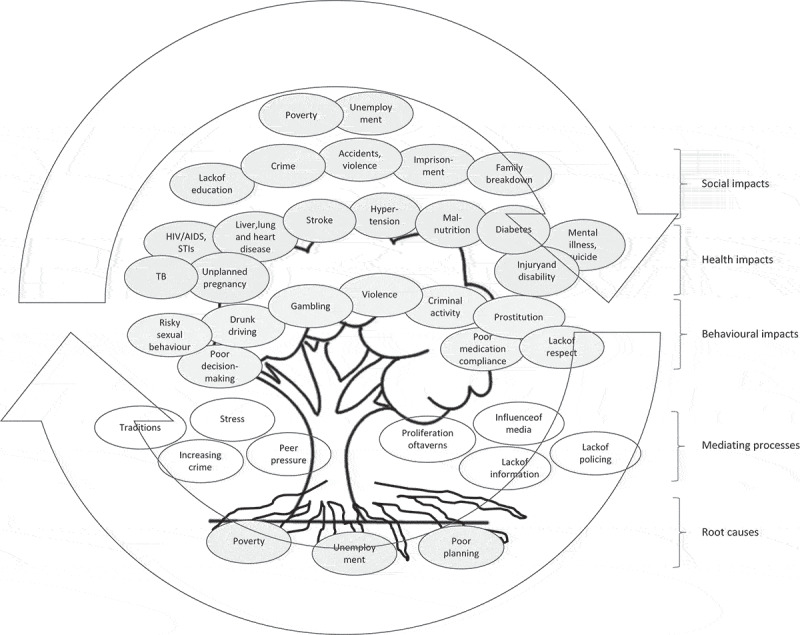
Problem tree developed by community stakeholders illustrating the non-linear, self-reciprocating relationships between causes and effects of AOD abuse

AOD abuse is a serious problem in South Africa. Post-apartheid a new political order was instituted promoting inclusive development to right past injustices. The health sector underwent fundamental transformation to a community-oriented system focused on equitable provision, prevention, and health promotion [4]. There was also the expansion of trade and improvements in air and sea travel. This, combined with the geographical position of the country, meant that the availability and affordability of AODs increased sharply, and rural, black communities ravaged by poverty and racial segregation borne of decades of oppression became conducive environments for abuse and dependency [5,6].

Today, South Africa has the highest rate of alcohol consumption in the southern African region [7,8]. About a third of adults report harmful use [6,8–10]. Alcohol abuse in adolescents is associated with risky sexual behaviours, academic failure, absenteeism, and increased risk of drug use [8]. In 2015, over 13,000 people died in road traffic accidents of which almost 60% were alcohol-related [11]. South Africa also has the highest prevalence of foetal alcohol spectrum disorder (FASD) globally [12]. Alcohol abuse is linked to domestic and interpersonal violence, intentional and unintentional accidents, and premature death [13,14]. Substance abuse is also widespread, with 15% of the population estimated to use drugs regularly [8,15]. Substance use is linked to many forms of crime and violence, suicide, HIV/AIDS, and premature death, particularly among youths [8,16–18].

While AOD policies in South Africa have historically been characterised by a prohibitionist and supply-reduction focus aimed at realising drug-free society, there have been progressive shifts recently. The *National Drug Master Plan (NDMP)* 2013–17, adopted a public health, rights-based, harm reduction focus, acknowledging the multiple structural drivers of exposure, risk, behaviour, and harms [19–23]. Forward-thinking policies have, however, been rendered ineffective by various structural and organisational challenges, and in 2016, the government reported no significant reductions had been achieved in substance abuse rates [24].

This research was based on the premise that involving affected communities in the development of responses to complex public health issues can contribute to acceptable, sustainable, equity-oriented responses [25,26]. While community participation is a key issue on national and international agendas, the concept remains poorly understood, especially at operational levels and communities grappling with AOD are rarely engaged in the development of responses [27–29]. Similarly, while research and intervention development are well-informed through processes involving researchers, professionals, and community members in problem definition, design, and analysis, AOD often is dealt with scientifically by quantitative methodology, where participation is not usually called for [30].

Our aim was to contribute new knowledge on how to operationalise participation to address complex public health issues in disadvantaged communities. The objectives were to: (1) document relevant contexts, forms, and processes of engaging people to systematise experience of priority health concerns into shared forms of knowledge and to collectively identify remedial actions; and (2) reflect on and adapt the process building participation in local health systems.

## Methodology

Participation is a political process that recognises and enables those at the heart of the issue to address it and learn from the process. Recognising that people’s chances of being healthy are affected by social structures and systems, the approach is not simply a target-oriented intervention, but is instrumental and substantive, an interchangeable means and end. As a result, participation is a contested concept with a range of interpretations.

Participation does not conform to conventional evaluation: both target-oriented and empowerment variants are unrealistic when informed by linear, causative assumptions, which deny dynamism and interaction through which the method is more correctly interpreted [31]. Contextualised documenting of process and iterative learning is therefore seen as more appropriate to identify and share practical experiences, and examine forms and process of participation in particular environments [32].

The research was thus rooted in constructivist and participatory enquiry paradigms [33–35], based on assumptions that practical, experiential knowledge that is co-constructed, self-reflective and embedded in complex, adaptive social and health systems can support and inform the organisation and delivery of equity-oriented and people-centred public services. Beneficiaries were defined as people, and health systems actors in the wider research programme (described below), in resource constrained systems who collectively share rights and responsibilities for health and health services.

## Methods

In partnership with community stakeholders, the research team progressed a Participatory Action Research (PAR) process to engage marginalised communities to identify and address local health concerns [36,37]. PAR seeks to enable and consolidate community resources to advance social justice, disrupting conventional subject-object research approaches, transforming the roles of those who usually participate as research subjects into active co-researchers and change agents. The PAR study was part of a programme entitled VAPAR (Verbal Autopsy with Participatory Action Research), connecting service users and providers to generate and act on research evidence of practical, local relevance. The wider programme is informed by views of health systems as complex, adaptive, human, and relational, in which communities and health workers have substantial knowledge of care processes and norms, and how health policy is ‘brought alive’ through these [26].

The analysis of the PAR process was informed by three main practice frameworks. Firstly, Arnstein’s classic ladder on forms and extents of citizen participation [38]. Secondly, a recent review of community mobilisation processes (mechanisms, enablers, and barriers), in which mechanisms are defined in instrumental terms as intervening between delivery of activities and outcomes, with enablers and barriers defined as features of the environments (physical and social), including process design, that modify ability to produce outcomes [39]. Thirdly, we drew on international learning on building social participation in health systems reflecting empowerment perspectives [40].

The research was progressed at the MRC/Wits Rural Public Health Transitions Research Unit (Agincourt) Health and Socio-Demographic Surveillance System (HDSS) study area located in Mpumalanga province, South Africa. The HDSS was established in 1992 to support the district health system with robust and timely data on vital aspects of people’s lives, namely births, deaths, and causes of death. The HDSS covers an area of 450 km^2^ with a population of about 120,000 occupying 21,500 households in 31 villages. Conditions in poor and rural villages are comparable with many settings in the region: there is limited piped water, rudimentary sanitation, underdeveloped roads, unaffordable electricity, and high unemployment [41]. The burden of HIV is high and is highly unequal. A population-based survey in the study area indicated 19.4% prevalence among people 15 and older [42]. Nationally, the prevalence in black populations is 40–50 times that of whites and in adolescents, risks are eight times higher in females than males [43].

PAR was progressed in three villages in the Agincourt HDSS study area. To sustain and develop relationships, the research team reconnected with participants involved in previous research with the group [44–46]. (The term *participant* refers to persons engaged in the process and is used synonymously with *community stakeholder*). In the prior work, twenty-four [24] participants had been recruited over three villages purposively to gain inputs from a cross-section of the community including traditional healers, community and religious leaders, community health volunteers, and family members. In one village, the participants were women of reproductive age only. The villages had similarly been purposively selected to vary by accessibility to health services and levels of child-headed households. From the original 24 participants, 13 agreed to be involved, and 11 individuals with characteristics commensurate with the previous recruitment criteria.

In each village, an introductory workshop was held. Researchers described intentions to share power and control between the researchers and participants, and inputs were sought from community stakeholders on topics on which the process should focus. Through facilitated discussions, participants nominated a range of priority health issues, which were prioritised using ranking and voting exercises. Facilitators also sought direction from participants on expanding the participant base to include relevant perspectives in the process. In two villages, AOD abuse was nominated as the most important health issue and youths (18–35 years) were identified as a group affected by, and whose views are often excluded from, action to address it. Through the same process, the third village nominated shortages of clean, safe water. (This paper reports on AOD abuse, the results on water are presented elsewhere [47]). On this basis, 16 additional participants, eight in each village, were identified by participants and recruited by researchers (Table 1). A series of workshops was then held over seven consecutive weeks through which participants shared and systematised experiences to build consensus on the problem, locally acceptable actions to address it, reflected on and refined the process, and discussed next steps. A total of 16 workshops was held. These are described in more detail below and are summarised in Table 2 and supplementary material 1.

**Table 1.** Composition of discussion groups

Participants*	Original	New	Total
*(a) Three village-based discussion groups (including group nominating water)*			
Religious leader	1		1
Traditional healer	3		3
Community official	5		5
Community health workers	3		3
Family member**	7	4	11
Woman of reproductive age	5	4	9
Youth		16	16
TOTAL	24	24	48
*(b) Two village-based discussion groups (group nominating AOD abuse)*			
Religious leader	1		1
Traditional healer	2		2
Community official	3		3
Community health workers	2		2
Family member**	4		4
Woman of reproductive age	4		4
Youth		16	16
TOTAL	16	16	32

*All participants were 18 years or older. Acknowledging that participants have multiple roles at home and in the community, a primary role was agreed with participants for the purposes of recruitment. **Close relatives: parents, siblings, in-laws, nieces, nephews, cousins.

**Table 2.** Schedule of workshops and PAR tools and techniques

Community-based group (priority health topic)	Focus topic (tools and techniques)							
	Week 1	Week 2	Week 3	Week 4	Week 5	Week 6	Week 7	Week 8
A (AOD)	Workshop 1: Topic selection (ranking and voting)	Workshop 4: Causes/ Impacts (problem tree)	Workshop 7: Impacts/Actors (Venn diagram)	Workshop 10: Action (action pathways)	Workshop 13: Causes/ Impacts (problem tree)	Workshop 14: Impacts/Actors (Venn diagram)	Workshop 15: Action (action pathways)	Workshop 16: Reflection and next steps (facilitated discussion)
B (AOD)	Workshop 2: Topic selection (ranking and voting)	Workshop 5: Causes/ Impacts (problem tree)	Workshop 8: Impacts/Actors (Venn diagram)	Workshop 11: Action (action pathways)				
C (Water)*	Workshop 3: Topic selection (ranking and voting)	Workshop 6: Causes/ Impacts (problem tree)	Workshop 9: Impacts/Actors (Venn diagram)	Workshop 12: Action (action pathways)				
Ranking and voting	To identify priority health topics of relevance to the community. A list of health priorities was developed during the discussion, after which participants voted for the topics of highest relevance using adhesive stickers. The voting progressed through two rounds with discussion and agreement at the end.							
Problem tree	To understand and ‘unpack’ nominated topics from different perspectives. Through facilitated discussions using a tree diagram visible to all, participants identified cause-and-effect relationships at various levels from root (tree roots) to intermediary causes (trunk and branches) and consequences and other effects (tree pods), building subjective perspectives into shared accounts through consensus.							
Venn diagrams	To understand impacts and actors involved. Collective account developed with Venn diagram made from cardboard circles of different sizes and colours to indicate relationships and interactions between various actors and institutions, identifying internal and external organisations active in the topic and how they related to one another in terms of contact and collaboration.							
Action pathways	To articulate overall goal(s) to address the issues identified and visualise and depict stepwise actions and actors to achieve these. The action pathway was collectively developed to represent moving towards a desired goal via a series of interconnected steps and events.							
Photovoice	To visually convey lived experience. Participants given basic training in photography, research ethics and digital cameras to take photographs illustrating the topic or condition as it existed in the physical environments. Photographs presented and discussed in meetings, and captions developed to describe what images conveyed.							
Facilitated discussion	On reflections and next steps: to reflect on experiences, outputs and how the process should be carried forward to engage government and non-government organisations. Participants discussed differences and similarities between the workshop outputs, cross-verified each other’s outputs and reflected on the process and future development							

* Results presented elsewhere. Source [69].

In the second week, participants shared knowledge and experiences of AOD abuse using a *problem tree* to identify, map, and classify cause-and-effect relationships. In the third week, actors and institutions with roles in, or responsibilities for, the issues, and their inter-relationships were mapped using a *Venn diagram* to initiate a discussion on action. These elements were drawn together in the fourth week in which participants developed *action agendas* to address issues identified. Between the fifth and seventh week, participants from all villages came together to validate and build further consensus on the topics and action agendas. In the final week, stakeholders reflected on the process and way forward.

A visual method called *Photovoice* was also used [48]. Participants were provided with digital cameras and invited to take photographs capturing aspects of physical environments relevant to the discussions. The research team provided basic training on photography, and on how to use the cameras and obtain permissions from subjects of the photographs. Photographs were presented in workshops from the third to eighth week, in which participants selected images to visually represent the issues being raised, and collectively developed descriptive captions.

The research team also provided information on the burden of mortality owing to AOD abuse among youth and adolescents using mortality data from the Agincourt HDSS (2012–16). We reviewed the main categories of cause of death and assigned likelihoods of relationship with AOD abuse as *very likely, likely, possible* and *probably not*. Mortality due to road traffic and other accidents, suicide, assault, HIV/AIDS and liver problems was ascribed as very likely, and deaths owing to non-communicable diseases, and pregnancy-related causes were ascribed as likely. According to this analysis, half the 216 deaths in the 12–24 years age group (corresponding to a mortality rate of 1.42 per 1,000 person-years) were classified as preventable had there been avoidance of AOD abuse.

The workshops were facilitated by the researchers (DM, with support from JH, RT, LD, and MV) and held in *XiTsonga* with some content in English, in sessions lasting 1.5–3 hours. As the series progressed and familiarity with the process grew, participants were invited to adopt more active roles, presenting and deliberating over the results and facilitating discussions. With separate permission, each session was audio recorded, and later transcribed and translated into English. Ten percent (10%) of transcripts was back-translated and checked for meaning and accuracy.

Analysis of narrative and visual data was conducted, to the extent possible, to preserve the community voice and illustrate the process and outputs generated by community stakeholders. Transcripts and visual data were thematically analysed. Researchers (LD, OO, DM, RT, JH) familiarized with the data, creating and assigning codes iteratively until no new codes emerged. Codes were then grouped into themes and sub-themes. Meanings, relationships, and contradictions within and between themes were noted [49]. The research team then framed the descriptive results with reference to practitioner frameworks of citizen participation [38], community mobilisation [39] and social participation in health systems [40].

Ethical conduct was considered in terms of control over the process and power dynamics among intended beneficiaries. Potential power imbalances were acknowledged, considered and re-visited throughout, and mutual respect for traditions, languages, and values of participants were ensured. Potential consequences (harmful and beneficial) were explained to all participants prior to involvement, and convenient locations were used for the workshops. Participants were informed that they could withdraw from the process at any time, and were provided with refreshments, transport costs and were reimbursed for time spent participating with 300ZAR (21USD) per participant. All identifiable data were anonymised. Data were stored on secure servers hosted by the Agincourt HDSS and the University of Aberdeen, and managed and analysed using NVivo version 12 [50]. The research team secured approval from Mpumalanga Health Research Committee (MP_201712_003) and the Research Ethics Committees of the University of Witwatersrand (M171050) and the University of Aberdeen (CERB/2017/4/1457).

## Results

We present the outputs and reflections generated by community stakeholders below illustrated by the thematic narrative and visual data analysis (supplementary material 2).

### Nominating the issue

The research team reconnected with community stakeholders, described the study and co-design intentions. Participants were positive about involvement and we worked together to identify priority health concerns. Through facilitated discussions, a long list of topics was developed, interrogated, and cross-verified. Given the volume of priory health concerns, two rounds of voting were held to identify priority issues (supplementary material 3). Following the nomination of AOD abuse, the discussions on additional relevant perspectives to include identified young people as particularly affected by, and typically excluded from, discussions on action to remedy the issue.

The problem that our community is faced with is alcohol and the drugs … that needs to be addressed to our children, the youth of our community because they are the ones consuming the alcohol and spending their time in taverns (older participant, workshop 2).

### Generating collective accounts

In the second week, engaging with larger groups of older and youth participants, we collectively identified and mapped causes, impacts, and mediating processes related to AOD abuse. Poverty and unemployment were identified as fundamental drivers, with poor planning due to perceived corruption in the authorities seen as having entrenched poverty and unemployment in communities. There was consensus that disenfranchised and disillusioned youths and adults visiting taverns to while away time are drawn into AOD abuse (supplementary material 4). School-leavers were seen as particularly vulnerable with many accounts of substance use and abuse among children to relieve stress from hardship and poverty, and of prostitution among women and girls.

Some children cannot take the stress they get from being poor that’s why they end up using drugs and alcohol as a stress reliever (youth participant; workshop 7).

The large and increasing number of outlets (taverns, bottle stores, and a relatively small number of unlicensed outlets) was repeatedly raised as a major contributory factor, with many reported to operate for long hours, some 24 hours, with noise pollution, safety concerns, gambling, prostitution, unsafe sex, and the sale of alcohol to children described. There were perceptions of tolerance for tavern operators breaking the law among corrupt law enforcement officers also in need of money.

… the taverns opens day and night and our children go there always. There are no rules to be followed. The police are the cause of that because they are in need of money … if the police … go to [name] tavern to talk to the tavern owner … he can bribe the police so that they should keep quiet (youth participant; workshop 4).

While there was consensus on many aspects of the issue, different perspectives were also evident. Older participants described the influence of television, mass media, and peer pressure, pointing out that the media frequently portrays AOD abuse as desirable and, without information on harms, perpetuates the crisis. Youth participants had complementary perspectives, describing AOD abuse as socially normal and necessary, describing perceived positive impacts on social status.

They broadcast people drinking alcohol and smoking different types of drugs … our children emulate the lifestyle hoping to be like them. In the end they lose their future (older participant; workshop 5).

… when you don’t smoke or drink you are not human (youth participant; workshop 5).

Older participants shared knowledge and experience of heavy AOD consumption in the home and at festivals, with potent home-brewed wine, beer, and the spirit (*Xipayoni*) (supplementary material 5,6). Youth participants shared further viewpoints that AOD use and abuse were common among people engaging in criminal activities to enable feelings of boldness and mask conscience, and reported marijuana (*dagga*), benzene, glue, and antiretrovirals (ARVs) being mixed and smoked (*Nyaope*) often among children (supplementary material 7). Further mediating processes identified included poor parenting, and a lack of information, resources, and recreational facilities for young people.

They also mix the pills [ARVs] to the things that they smoke (older participant; workshop 4).

A range of health impacts were identified hypertension, stroke, cancer, diabetes, other heart, liver and lung disease, mental illness, suicide, malnutrition, HIV/AIDS, STIs, TB, unwanted pregnancy, accidents, injuries, and disabilities. Behavioural impacts included impaired decision-making ability, risky sexual behaviour, non-adherence to medications, poor nutrition, drinking and driving, gambling, crime, violence, including sexual violence, prostitution, and addiction (supplementary material 8). Many older participants expressed distress caused by disrespect for traditional values of modesty and respect for self and others among children and youths abusing AODs, which often had social consequences for parents and families.

I bought nice dresses, but my son took them and sold them so that he could get money to buy drugs … some of the community members said that I am also involved because they think he shares the money with me after he sells the things that he stole. It is painful and it affects me a lot (older participant; workshop 4).

Participants consistently described the cumulative effects of AOD abuse as *destructive* of families and communities, identifying children and youth as groups disproportionately affected. Participants noted episodes of domestic violence in homes where parents are often intoxicated, with family breakdown, separation, divorce, and suicide often conveyed. Further social impacts were identified and mapped on education and employment, violence in taverns, road safety, crime, and imprisonment. There were many discussions on the causal directions of these relationships, which eventually settled on a reciprocal, self-sustaining relationship between causes and consequences (Figure 1).

… if we continue like this our children will not have a better future; our level of education will remain poor forever (older participant; workshop 5).

### Developing action agendas

As the series of workshops progressed, the discussions focussed on addressing the problems identified. In the third week, the workshops were configured to facilitate the identification and mapping of stakeholders with responsibilities for and towards addressing AOD abuse and its associated causes and problems (supplementary material 9). By this point, participants were more familiar with an interactive process focussed on community knowledge for action and learning, and responded well to the Venn diagram activities developing a shared visual representation of key actors and institutions with whom action could be progressed.

**Figure 2.**
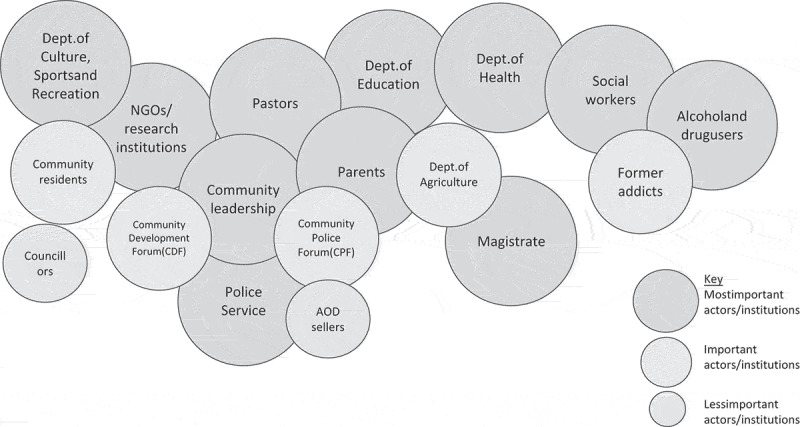
Venn diagram prepared by community stakeholders showing the range of actors addressing AOD abuse, and their levels of importance and connection to the community

The Venn diagrams depicted local community leaders as having central roles with a range of connections to parents, religious leaders, the police force, NGOs, and research institutions. Further capacity and resource were identified in existing community-based structures: the Community Development Forum (CDF), the Community Police Forum (CPF), and among community residents and former addicts. State actors identified included Department of Education (DoE), Department of Health (DoH), Department of Social Development (DSD), Department of Culture, Sports and Recreation, the South African Police Service (SAPS), and Mpumalanga Economic Regulator (former liquor board) (Figure 2).

**Figure 3.**
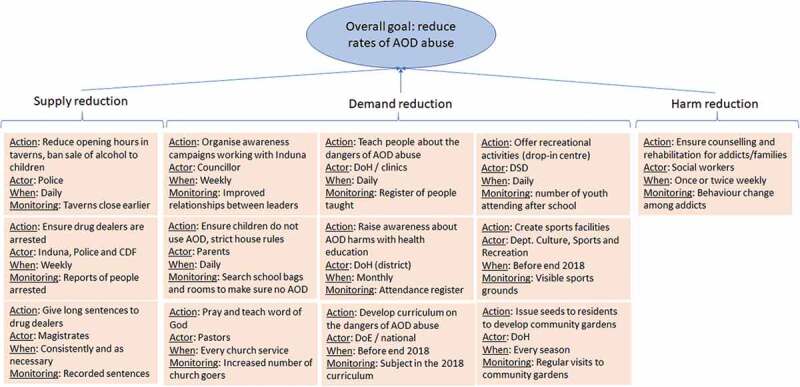
Action agenda prepared by community stakeholders to address AOD abuse in communities indicating overall goals, actions, actors, timelines, and progress monitoring

In the fourth week, the problem tree and Venn diagram were drawn together to develop action agendas. These defined overall goals, and stepwise actions and associated actors, timescales, and monitoring mechanisms through which goals could be achieved. Acknowledging the extent of the problem, an overall goal to decrease, rather than eradicate, AOD abuse was articulated and from which the action agendas were developed (Figure 3). Collective responsibilities for the action agenda items were also articulated and appraised throughout. The resulting action agendas were detailed, specific and integrated with actions arranged into demand, supply and harm reduction. These are described further below.

In terms of supply reduction, improved regulation of taverns was discussed, with various measures to encourage adherence to age restrictions, reduced opening hours, reduced attractions for youth, regular police inspections, and restrictions on opening taverns near to schools. Collective responsibility was highlighted. In the discussions, traditional leaders (*Indunas*) were seen as authoritative figures to promote compliance for regulations with tavern operators as an effective way to progress dialogue and cooperation, and with community surveillance to complement efforts of the CPF and police. A stronger police presence and tougher sentencing were also recommended.

We can involve the Induna … he should talk to the tavern owners to solve the issue … they should plan the time of opening the tavern and closing (youth participant; workshop 4).

In terms of demand reduction, the value and feasibility of awareness campaigns delivered by community leaders, educators, local authorities, and clinics with reinforcing activities in the home and faith-based organisations were discussed. Participants recommended that curricula should orient learners to the dangers of AOD abuse, engaging ex-drug abusers to provide real-life accounts. Children and young adults leaving school with limited employment opportunities were seen as a particularly vulnerable group. Support for school leavers with skill acquisition centres to improve employability, and drop-in centres to provide meaningful activities were highlighted as important. The action agendas included teachers and parents working together, and for teachers to monitor learners for AODs possession in schools.

The school needs to also work with parents … if there are challenges parents should be involved in solving or coming up with solutions (older participant; workshop 10).

Acknowledging that AOD abuse cannot be eliminated, participants made recommendations to reduce harms including psychological support for addicts delivered in communities. This was accompanied by recommendations to provide rehabilitation services and facilities to re-integrate recovered and recovering addicts into the community.

We want to see a rehab centre built in our community and more employment opportunities, these will help to achieve our goal (older participant, workshop 10).

### Reflecting on process

Participants reflected on the process in each workshop and in a dedicated workshop at the end of the series. The problem tree was well received and understood, and the multi-level interpretations developed were seen as useful information for the authorities on the extent and nature of the problem. The Venn diagrams were more challenging owing to the multiplicity of actors and viewpoints on the importance of and relationships between different actors. Nevertheless, participants developed organised and systematic ways to discuss and deliberate over the issues as a result:

… we should talk about one actor at a time because others talk about the pastor while some talk about police and it is confusing (youth participant, workshop 11).

In reflections on developing action agendas, there was a clear acknowledgement of the need for communities to be active change agents rather than passive beneficiaries of external programmes developed and run by other groups. With reference to the action agenda developed, participants acknowledged that community input and cooperation are necessary for programmes, resources, and other activities supported by the Agincourt HDSS (Wits), government departments, and NGOs to be successful.

… [in the action agenda] we have department of agriculture working with youth to create employment through creation of vegetable gardens. Wits [Agincourt HDSS] works with community leadership by doing research within the community. We have department of health that works with social workers to assist those that are damaged by drugs to get medical care, social workers offer counselling to the addicts and their families and send them to rehab centres for further management. Victims work with department of education to teach learners about bad effects of drugs and alcohol. We have a drop-in centre where youth will go to do activities that will distract them from drinking alcohol and using drugs. All these will never be successful without the cooperation of the people within the community (youth participant, workshop 14).

Community stakeholders acknowledged the opportunity to gain and share new knowledge, and appreciated taking active roles during the workshops, presenting findings and developing skills in communication, planning, public speaking, and photography (supplementary material 10, 11). They also acknowledged the potential for benefits to their communities and expressed expectations related to these. Expectations required sensitive and responsible management, with clear and open discussion around when, and with whom, action would occur.

Participant: … Aren’t you already sending what we are discussing to the relevant department? I mean if you are already updating them we expect something to be done by the end of this year.

Facilitator: VAPAR is a process, it is not something that we do and finish here. Like I said earlier, from next week we will be coming together with other villages, those villages have their own health topic. At the end we will analyse information from all villages before we include other departments (youth participant/VAPAR Researcher, workshop 10)

Some dissatisfaction was expressed regarding the levels of reimbursement and over cameras returned at the end of the process, which were seen as rewards for participating. Overall, however, participants were satisfied that they were consulted about how to address priority issues in their communities and looked forward to future collaborations. They also recognised and appreciated the relationships with co-researchers. Considering the next steps in which the evidence generated would progress into dialogue with the authorities, participants waived anonymity, agreeing to be acknowledged as photographers in the reproduction of visual evidence. Finally, at the end of the series, participants suggested having a celebration of the work, which was supported and encouraged, marking a positive end to the PAR component of the programme.

… this research will improve our community and we gain knowledge, we learned about caring for ourselves and to work together with other people. We are satisfied with the relationship that we have with people from Wits [Agincourt HDSS] (older participant, workshop 16).

## Discussion

In this section, we reflect on the contexts, forms, and processes of participation and elicit learning and adaptions to build participation in the local health system (Table 3, supplementary material 12).

**Table 3.** International learning on building social participation in health systems, process reflections, and future adaptions. Adapted from [40]

International learning	Process reflections	Adaptations
1. Participation is both a means for health improvement and an end in itself based on values and rights	Broader understanding of forms, processes and contexts requires explicit reference to, and ultimately transfer of power towards, disadvantaged groups, and a focus on change processes developing community voice as a continuous process, situated within social and political environments	Framing in terms of social justice and citizenship may help to communicate key features of the process and what it seeks to achieve
2. Community experience is a key entry point, and community activism and leadership are key drivers of participatory practice	Mobilisation activities related to participation and deliberation were possible to progress, and there was some evidence of individual acts of information sharing in the wider community	Enabling community experience and leadership, with less researcher control and more shared ownership should be incorporated into collective design decisions
3. Participatory processes and social power in health are more likely to thrive when services go into community settings	All activities were conducted in accessible community spaces in which there were generally supportive attitudes. Community settings appear supportive and enabling of PAR	While institutional and political support is important, *claimed* spaces in which issues can be autonomously raised and framed are important to cultivate and maintain
4. They are supported by and elicit more holistic models of health	Self-nominated priority topics, and facilitated participatory problematization clearly elicited holistic models of health	For acting on the evidence generated, a wider set of stakeholders should be engaged, beyond department of heath
5. Informal and formal spaces and processes both play key roles. The synergies and links between them enrich both	Formal (e.g. clinic committees) and informal (e.g. VAPAR) structures exist for community participation in this setting	Interaction between claimed and invited participatory spaces will be sought and progressed
6. Institutional and individual facilitators play a critical role	Sensitive facilitation was key to convey process, co-design, and power dimensions that enabled rich action-oriented interpretations of community nominated. Management of expectations important	Lift up and make explicit the key contribution of facilitation. Explore skills exchange for effective and respectful facilitation
7. Sharing Information and participatory processes to gather, analyse, discuss and use community evidence in planning are necessary (but not sufficient) for meaningful social participation	The wider VAPAR process is geared towards cooperative action cooperation with health authorities in the province, district, sub-district and locally	A wider set of stakeholders beyond department of health, should be engaged to share, interpret, act on, and learn from community evidence
8. Accessible processes for co-determination that link decisions to shared plans, actions and resources to act are central to meaningful participation		Careful consideration and appraisal of implications of proposed actions in cooperation with health systems stakeholders, and other government and non-governmental stakeholders, are necessary as process progresses
9. Deepening of participation takes a consistency of presence, time and capacities	The wider VAPAR programme supports this consistency	Attention to specifics of engagement over time, and beyond defined periods of engagement, is required with a focus on making implicit issues of presence, time and capacities explicit. Careful attention to issues of marginalisation and representation are required
10. Learning from action (and evaluation) needs to track diverse forms of progress to build strategic review	Wider VAPAR programme enables the tracking of progress	Diverse forms of progress (and failure) require careful monitoring as the action elements progress

The research team initially re-engaged with community stakeholders to prioritise prior relationships and learning. Participants were willing to be involved and were active and engaged, advising on priority health topics and expansion of the participant base. Thereafter, the wider and partly self-selected community stakeholder group contributed consistently in the weekly workshops, developing sophisticated accounts of relevant issues. There were some challenges related to expectations that required careful management and transparent, consistent communication. Otherwise and arguably related to the nature of the topic selected, some youth participants were dominant and at times disruptive. Through consistent and careful facilitation, however, more reserved participants were prompted to contribute, which led to more balanced participation overall.

Considering forms and extents of participation, the process as it was initiated can be considered a comprehensive *consultation* representing collective voices of community stakeholders on self-selected topics. The consultation was pragmatic and exploratory, demonstrating the potential to identify locally relevant issues and provide comprehensive renditions on the scale and nature of the problem. Community stakeholders’ accounts of AOD abuse were sophisticated extending linear notions of cause and biomedical individualism, and reflecting chronic AOD abuse, with adults and youth locked in cycles of abuse with destructive effects on individuals, families, and communities. While the findings support research on harms and shortfalls in policy and services [51,52], they also suggest that community control over problem definition allows relevant issues to be raised and framed in more complete ways than might be possible otherwise.

The process was configured to move from diagnostics to intervention, with youth and older participants’ perspectives brought together into shared action agendas. While complex and challenging to discuss and record, mapping strategic alliances among service providers, local authorities, community bodies, and individuals enabled a shift in the discussions, from relatively passive information-sharing towards the development of shared agendas, and on the relevant forms and extents of power to advance these. This shift also appeared to overcome dissatisfaction expressed towards the authorities over perceived corruption and poor planning, and reflected willingness to work cooperatively to consolidate resources and advance solutions. This reflects a transition from *consultation* towards a willingness to enter into fuller *partnerships* [38]. The findings also indicate that as the process continues, stakeholders should be engaged beyond the department of health to interpret, plan, act on, and learn from, community evidence.

The action agendas were however based on an assumed feasibility and readiness of the government to come to the table to address deeply contested social problems, raising broader issues of statutory versus individual and community responsibility within wider social, political and health systems contexts. In post-apartheid South Africa, there were radical advances towards community-based PHC and community participation [4,53–57]. While participation was mandated in many strategies and policies, it has been poorly understood and unevenly operationalised [58]. Progressive policies faced further challenges related to public sector underinvestment, human resource crises, corruption, poor stewardship, and deepening social and health inequalities [59–61].

Despite these challenges, the PHC re-engineering strategy launched in 2012 is a significant revival of the bringing of services closer to people, and similarly of engaging people in health and health care [62]. The strategy is part of a broader commitment in South Africa to National Health Insurance (NHI), which will necessitate fundamental systems change based on principles of universality and solidarity [56]. These require devolved operational management to organise resources based on local needs and priorities [63]. Recent policy and strategy shifts may therefore provide opportunities for cooperative learning processes inclusive of citizen voice.

Nevertheless, considering institutional challenges and realities, it may be pragmatic to build participatory processes and capacities through flexible and inclusive community spaces, and to consolidate and develop these as a basis from which to engage and develop relationships with other stakeholders and decision-makers [64]. Otherwise in terms of responsibilities, elements of the action agendas, e.g. community-based monitoring of taverns, could expose community actors to undue risk for which they are neither trained nor equipped. This also raises the need to connect with government departments and other stakeholders. As the process continues, careful consideration of the implications of proposed actions in cooperation with others is necessary.

Considering community mobilisation mechanisms [39], the process was designed to transfer power to those most directly affected to coproduce new knowledge for action with activities enabling group participation and deliberation. There were some individual acts of information sharing within the group, and suggestions of wider sharing in the community. Informal social support and collective action however were neither progressed nor evident in this element.

In terms of mediating capacities, efforts were made to democratise the process, ensure participation and local relevance, and collectively reflect on the power and fair benefit throughout. While the tools and techniques were developed with careful reference to methodological and theoretical debates, they were prepared in advance by the research team and arguably imposed on participants with little change or adaptation. In addition, while the results provide a granular account of lived experience of AOD abuse, rigorously verified at the community level and codified in detailed action agendas, dissemination and next steps with health and social services and non-state actors have similarly largely been driven by the research team (47). While significant control has been retained by the researchers, shifts towards the assuming of power and control were achieved. Enabling community leadership and shared ownership should be incorporated into collective design decisions as the progress continues to deepen participation and sustain relationships.

Otherwise, improvement of practical knowledge and skills was directly reported by participants, and there was some progress evidenced in participants’ reflections towards enabling critical consciousness, collective attitudes to AOD abuse, agency, and self-concept. There was also some progress in developing collective capacities, reflected in the willingness and commitment to continue the process, and reflected in the integrated action agendas. As described above, as the process shifted from problematising the issue, to strategically considering how and with whom action might be progressed to address it, support for and commitment to collective efficacy were expressed more explicitly as community stakeholders developed plans about how to use existing community structures and other assets to address shared problems.

In terms of enablers and barriers in community contexts and considering pre-existing conditions of poverty and social cohesion, attention to specifics of engagement over time (including in-between specific periods of engagement) is required. Also, in the context of the process, incentives were an important issue. While the researchers attempted to manage expectations, issues of material compensation required careful management, especially regarding implications of in-kind reimbursement, here with the digital cameras. Recognition of time inputs to the process is also important. While not all participants attended all workshops for the entire time, there was probably a core contact time of 12–15 hours per participant (not including travel and waiting times). In this regard, the reimbursement, with refreshments and refunds of travel costs, is probably comparable only to minimum wage. Careful consideration of the value of people’s time is required in future.

Otherwise, in terms of implementation, there was respect for culture and tradition. While English was used in many exchanges, the majority of the workshop discussions were held in local languages. The workshops were held in accessible community spaces, deliberating over community-nominated topics with community-directed recruitment of less powerful subpopulations. Nevertheless, the representativeness of participants requires ongoing consideration: 30% of the population in the Agincourt HDSS study area are former Mozambican refugees, a small proportion of whom remain undocumented and still suffer social exclusion. As the process develops, issues of marginalisation and representation, whether and on what basis to extend to other communities, and the implications for and roles of existing participants should feature in codesign discussions.

International evidence on building social participation in health systems indicates that facilitators have key roles as enablers and catalysts [40]. In our process, facilitators were authentic, trustworthy, had empathy for participants and their situations, backgrounds in PAR and community work, and professional experience in the health system including the community health system. These were further informed and enabled by the institutional base. The Agincourt HDSS has supported the health system for over 25 years and occupies a strategically important role as a legitimate, independent arbiter between rural voices and the district health system (www.agincourt.co.za). More generally, the government recently supported the consolidation and expansion of HDSS infrastructure to support evidence-informed public policy (http://saprin.mrc.ac.za/). Sustainability and transferability can thus be considered in terms of methodological development for the transition into routine health systems and demographic surveillance processes.

The next steps of the process, related to institutional linkages, is an important component of this. Involving communities in the design and monitoring of health services requires the overcoming of prejudices and establishing awareness, communication, and mutual respect. When situated in a medical or health paradigm, additional power asymmetries may further obscure an already contested approach [32,65,66]. Framing in terms of social accountability and citizen voice may help to communicate purpose and process around developing shared interests in and commitments to communities and district health systems cooperating towards improved decision-making [67].

Moreover, international evidence on social participation in health systems indicates that participation grounded in community settings and centred on community needs and voices is critical for sustainability. While institutional and political support is important, spaces in which issues can be autonomously raised and framed are imperative to cultivate and maintain. International evidence further indicates that both informal (*claimed*) and formal (*invited*) spaces and processes can be enriched through two-way exchange [40].

We can therefore identify processes, actors, and alliances to support participation addressing local priorities acknowledging: inherent dynamism; that community needs and priorities must be articulated, and may not necessarily be in terms of healthcare; and acknowledging the central role of power. Ongoing attention and efforts are required to sustain community and institutional relationships, consider power and voice, and how, and with whom, to maintain the participative space. International learning on social participation in health systems indicates that diverse forms of progress (and failure) require careful monitoring as the action elements progress (Table 3).

Fundamentally, PAR is concerned with action and learning from action where there is an imperative and commitment to progress the action agendas developed. As described above, the PAR was part of a wider programme (VAPAR, www.vapar.org), expanding the knowledge base through partnerships for action on health equity. The results of the process have been further analysed and interpreted with health planners and managers at district and provincial levels and community stakeholders, and from which shared action plans have been developed and implemented [68].

## Conclusion

We report on the forms, processes, and contexts of participation from a research programme helping communities and district health systems to cooperatively improve services and decision-making. In the initial community-based element, we find significant willingness and capacity among community stakeholders to work in partnership with the authorities to address priority health concerns. In terms of learning, adapting, and building the process, flexible and inclusive spaces can build confidence and capacity among those most directly affected as a basis from which to raise and frame issues, which may help to inform action to enhance service delivery and, more generally, encourage shared responsibility for health with decision-makers and other stakeholders. More generally, broader understandings of participation require explicit reference to, and ultimately transfer of power towards, those most directly affected. Our learning and adaptation prioritise flexible and inclusive processes, developing community voice as continuous processes within social and political environments. We seek to further expand and deepen these foundations by engaging and forming new and wider alliances among service providers, planners, and other decision-makers.

## Supplementary Material

Supplemental Material

## Data Availability

Data available on reasonable request to the corresponding author.

## Paper context

Community participation can improve service responsiveness and outcomes. However, there is a lack of understanding of the concept. Adopting a participatory action research (PAR) process, the paper documents the inputs, contexts, actors, enablers, and challenges in gaining community voices on self-nominated priority topics. Flexible and inclusive participatory spaces can build confidence and capacity among those most directly affected, as a necessary basis from which to frame issues and develop solutions with decision-makers and other stakeholders.
