# Supplementary material for: Building cooperative learning to address alcohol and other drug abuse in Mpumalanga, South Africa: a participatory action research process
Source: Glob Health Action. 2020 Mar 2;13(1):1726722. doi: 10.1080/16549716.2020.1726722 (PMC7067166; doi:10.1080/16549716.2020.1726722)
Supplement: Supplemental Material [file ZGHA_A_1726722_SM2442.zip › Supplementary material_01_Flowchart.pptx]

## Slide 1
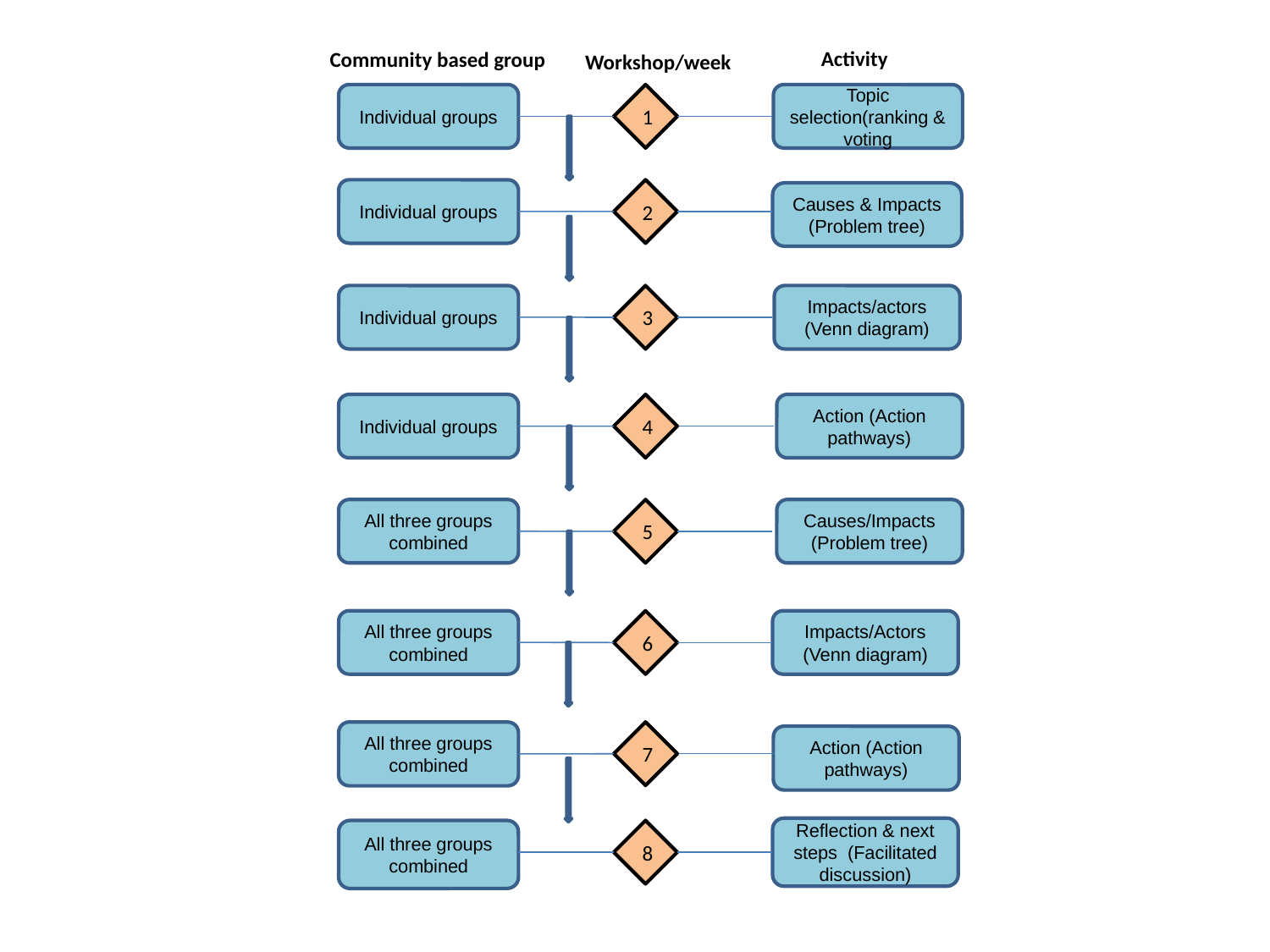

Activity
Workshop/week
# Community based group
Individual groups
1
Topic selection(ranking & voting
Individual groups
2
Causes & Impacts (Problem tree)
Individual groups
3
Impacts/actors (Venn diagram)
Individual groups
4
Action (Action pathways)
All three groups combined
5
Causes/Impacts (Problem tree)
All three groups combined
6
Impacts/Actors (Venn diagram)
All three groups combined
7
Action (Action pathways)
Reflection & next steps (Facilitated discussion)
All three groups combined
8
