# Supplementary material for: Building cooperative learning to address alcohol and other drug abuse in Mpumalanga, South Africa: a participatory action research process
Source: Glob Health Action. 2020 Mar 2;13(1):1726722. doi: 10.1080/16549716.2020.1726722 (PMC7067166; doi:10.1080/16549716.2020.1726722)
Supplement: Supplemental Material [file ZGHA_A_1726722_SM2442.zip › Supplementary material_02_Thematic framework.docx]

Supplementary material 3: Thematic framework

| FOCUS | THEME | SUB-THEME | |
| --- | --- | --- | --- |
| CAUSES | Root causes | Poor leadership, corruption | |
|  |  | Unemployment | |
|  |  | Poverty | |
|  | Intermediate causes | Proliferation of taverns, closing hours, sale of alcohol to children | |
|  |  | Lack of information on harms, rehabilitation and recreation | |
|  |  | Peer pressure | |
|  |  | Negative influence of media | |
|  |  | Poor parenting, family breakdown | |
|  |  | Traditional practices | |
|  |  | Lack of policing, corruption among law enforcers | |
|  |  | Increasing crime rate, including prostitution | |
| IMPACTS | Behavioural | Impaired decision-making e.g. drinking and driving | |
|  |  | Risky sexual behaviour | |
|  |  | Criminal behaviour | |
|  |  | Violence including sexual violence | |
|  |  | Prostitution | |
|  |  | Addiction | |
|  |  | Poor compliance with medications | |
|  |  | Poor appetite | |
|  |  | Loss of dignity | |
|  |  | Disrespect for parents and elders | |
|  | Health | Chronic conditions e.g. liver, lung and heart disease, cancers, stroke | |
|  |  | HIV/AIDS, TB and STIs | |
|  |  | Mental illness, depression, suicide | |
|  |  | Teenage/unwanted pregnancy | |
|  |  | Malnutrition, hypertension, stress | |
|  |  | Accidents, injuries, disability | |
|  | Social | Destruction of families and communities | |
|  |  | Domestic violence, divorce, family breakdown | |
|  |  | Increased threat of accidents, physical and sexual violence | |
|  |  | Increased school dropout | |
|  |  | Increased crime and imprisonment | |
|  |  | Increased unemployment and poverty | |
| ACTIONS | Demand reduction | Community | Awareness campaigns by CDF/ex-addicts |
|  |  |  | Improved and co-parenting |
|  |  | Government | DoH/DoE organize awareness campaigns |
|  |  |  | DoE introduce AOD abuse in school curriculum |
|  |  |  | DoE monitor learners for drugs in schools |
|  |  |  | DSD drop-in, vocational, skill acquisition centres |
|  |  |  | DoA support youths to venture into farming |
|  | Supply reduction | Community | Surveillance of taverns by CPF/community members |
|  |  |  | Dialogue with tavern operators on regulations |
|  |  | Government | Enforcement of laws regulating taverns by police |
|  |  |  | Prosecution/punishment for law-breaking tavern owners |
|  | Harm reduction | Community | Psychological/spiritual support from religious leaders |
|  |  | Government | Construction of rehabilitation centres |
| PROCESS | Benefits | Gained knowledge and new skills | |
|  |  | Process has potential for addressing AOD challenge | |
|  |  | Built relationships in communities and with research team | |
|  | Drawbacks | Reimbursement could be greater | |

CDF: Community Development Forum; CPF: Community Police Forum; DoH: Department of Health; DoE: Department of Education; DoA: Department of Agriculture; DSD: Department of Social Development
