# Supplementary material for: Building cooperative learning to address alcohol and other drug abuse in Mpumalanga, South Africa: a participatory action research process
Source: Glob Health Action. 2020 Mar 2;13(1):1726722. doi: 10.1080/16549716.2020.1726722 (PMC7067166; doi:10.1080/16549716.2020.1726722)
Supplement: Supplemental Material [file ZGHA_A_1726722_SM2442.zip › Supplementary material_12_Mechanisms.docx]

Supplementary material 13a: Possible mechanisms of community mobilisation and extent of achievement. Adapted from: (40)

| **Possible mechanisms** | **Description** | **Achieved** | **Partially achieved** | **Not achieved** |
| --- | --- | --- | --- | --- |
| **Mobilisation activities** | | | | |
| Group participation | Community members attend group meetings and become members of their community group | x |  |  |
| Group deliberation | Group members engage in open, critical dialogue with each other and their facilitator, identify shared problems, decide on and set goals, develop collective solutions and evaluate past initiatives | x |  |  |
| Individual acts of information sharing | Sharing information within the group and across social networks in the wider community |  | x |  |
| Informal social support | Mutual emotional, economic and practical support; referral for health problems; crisis support and protection from violence and harassment |  |  | x |
| Collective action | Group and community members carry out collective action to address shared health issues, such as protest, self-help or resource mobilisation |  |  | x |
| **Mediating capacities** | | | | |
| **Individual level** |  |  |  |  |
| Practical knowledge/skills | Leadership, negotiation and communication skills; problem formulation, decision-making and problem-solving skills; ability to translate theory into action | x |  |  |
| Critical consciousness | Capacity to critically examine one’s own and others' beliefs and values, relate one’s own vulnerability to wider social forces and question the immutability of everyday reality |  | x |  |
| Attitudes and norms relevant to a health issue | Concern for a health problem; perceived value of addressing a health problem; perceived social disapproval of harmful behaviour; critical personal attitude to harmful behaviour |  | x |  |
| Self-concept | A sense of agency, purpose and inspiration in one's own life; a sense of confidence and self-efficacy; self-worth and self-esteem; a sense of entitlement to basic rights; improved self-knowledge |  | x |  |
| Technical knowledge/skills | Knowledge of the epidemiology of a health problem, knowledge of effective ways to address it, knowledge of legal rights and entitlements |  | x |  |
| **Household level*** |  |  |  |  |
| Women’s position in the household | Status, respect, support and decision-making power in the household for women |  | n/a |  |
| **Collective level** |  |  |  |  |
| Civic attitudes and norms | Shared attitudes and norms around informal social support and collective action; shared belief in the collective efficacy of one’s group or community | x |  |  |
| Institutional linkage | Dialogue and partnership between community and institutions; better accountability and responsiveness of institutions to the community; links between community groups and institutions | x |  |  |
| Self-governance | Sense of ownership over process of addressing a health issue; presence of initiative and leadership; effective management of own resources; ability to discuss, agree and make decisions as a group |  | x |  |
| Social cohesion | A shared sense of belonging, identity and trust; well connected, mutually supportive social networks; cohesion among group or community members |  | x |  |

* Gram work on processes of community mobilisation is based on work around maternal and child health, as such while mediating capacities at household level are important to consider, women’s status in the household was not a focus progressed in this work

Supplementary material 13a: Enablers and barriers to community mobilisation and extent of existence Adapted from: (40)

| Proposed enablers and barriers | | Exists | Partially exists | Does not exist |
| --- | --- | --- | --- | --- |
| **Community context** | **Description** |  |  |  |
| Pre-existing poverty | Material poverty, poor access to employment and education, financial dependence on husbands or employers, insecure tenure of housing | x |  |  |
| Pre-existing social cohesion | Existing sense of belonging, identity and trust, existing social networks and community groups, history of living and working together | x |  |  |
| Supportive institutional-political context | Political will to tackle health issue, health system minimally functioning and able to respond to community concerns, lack of violent conflict, insecurity and instability |  | x |  |
| Supportive pre-existing health beliefs, attitudes and norms | Existing awareness and concern with health issue, prior confidence that issue can be addressed, culture of open discussion around issue |  | x |  |
| Pre-existing power hierarchies in the community | Lack of voice and decision-making power for women in the community, stigmas of sex and reproduction, power relations between men |  | x |  |
| Pre-existing power hierarchies within households | General lack of female household agency; husbands forbidding wives to attend group meetings; unequal power relations between daughters-in-law and mothers-in-law |  | x |  |
| **Intervention context** | **Description** |  |  |  |
| Intervention design and management | | | | |
| Staff management | Effective recruitment, training and supervision of group facilitators; staff confidence, motivation and retention | x |  |  |
| Incentives for participation | Cash or food transfers at group meetings; reimbursements for taxi fare; microfinance initiatives; help accessing entitlements | x |  |  |
| Managing community relations | Engaging stakeholders; avoiding backlash; building relationships with community members | x |  |  |
| Intervention implementation | | | | |
| Respect for local people, knowledge and practices | Avoiding trying to ‘teach’ group members and being open to learning from group members; negotiating flexibly, not demanding change | x |  |  |
| Relevant education tools | Locally accessible education materials; relevant language used; presence of a meeting agenda | x |  |  |
| Inclusion of less powerful subpopulations | Participation of less powerful community members and equal opportunity for all to contribute to group activities | x |  |  |
