# Supplementary figures and images for: Building cooperative learning to address alcohol and other drug abuse in Mpumalanga, South Africa: a participatory action research process
Source: Glob Health Action. 2020 Mar 2;13(1):1726722. doi: 10.1080/16549716.2020.1726722 (PMC7067166; doi:10.1080/16549716.2020.1726722)

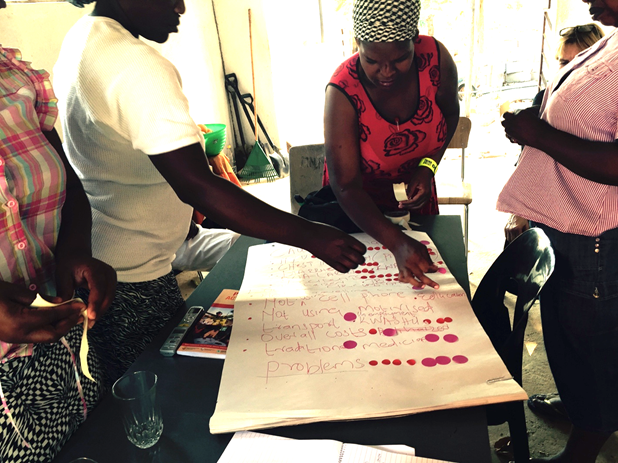

Supplement: Supplemental Material [file ZGHA_A_1726722_SM2442.zip › Supplementary material_03_Nominating issue.png]

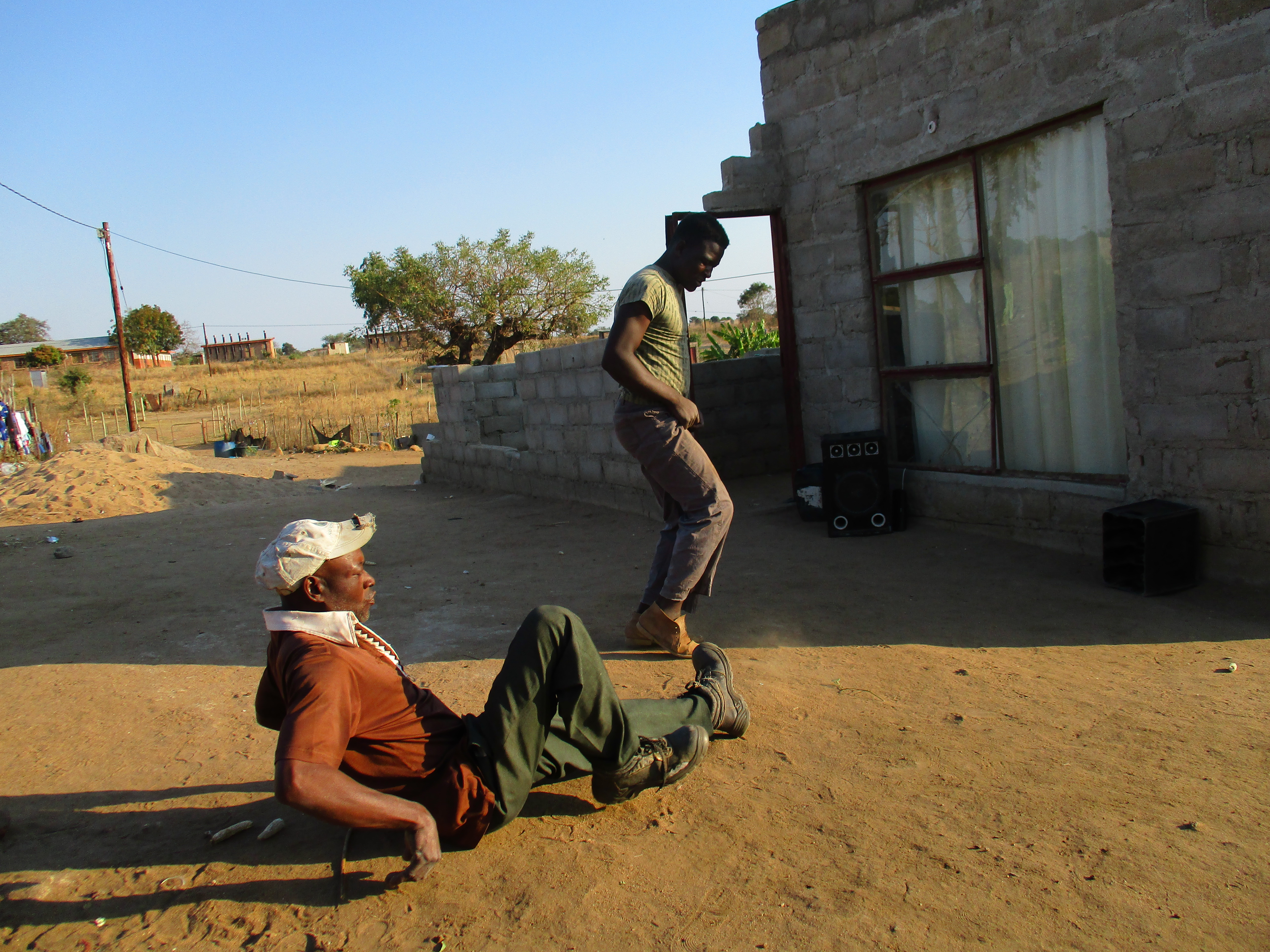

Supplement: Supplemental Material [file ZGHA_A_1726722_SM2442.zip › Supplementary material_04_Stress.JPG]

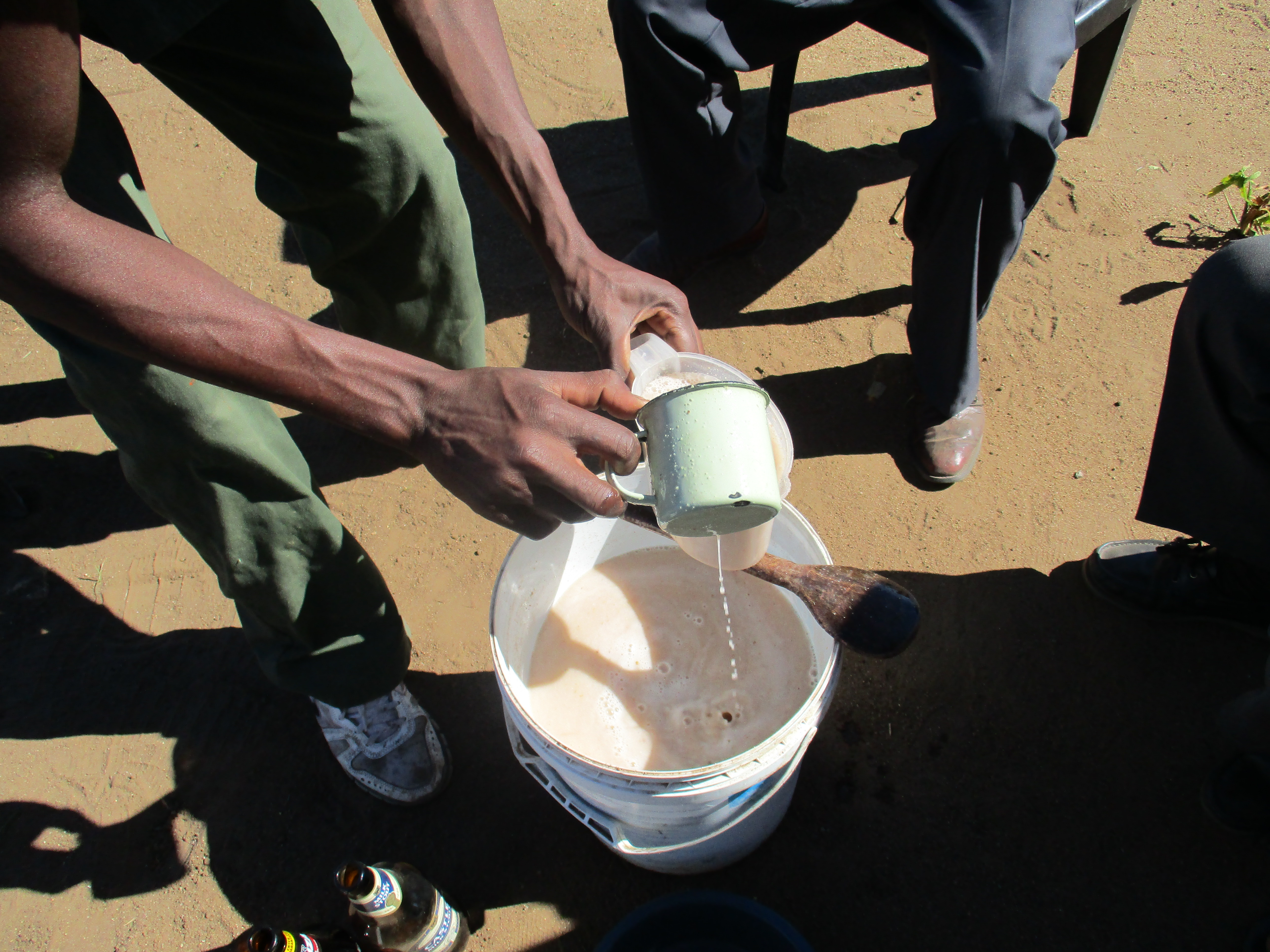

Supplement: Supplemental Material [file ZGHA_A_1726722_SM2442.zip › Supplementary material_05_Trad beer.JPG]

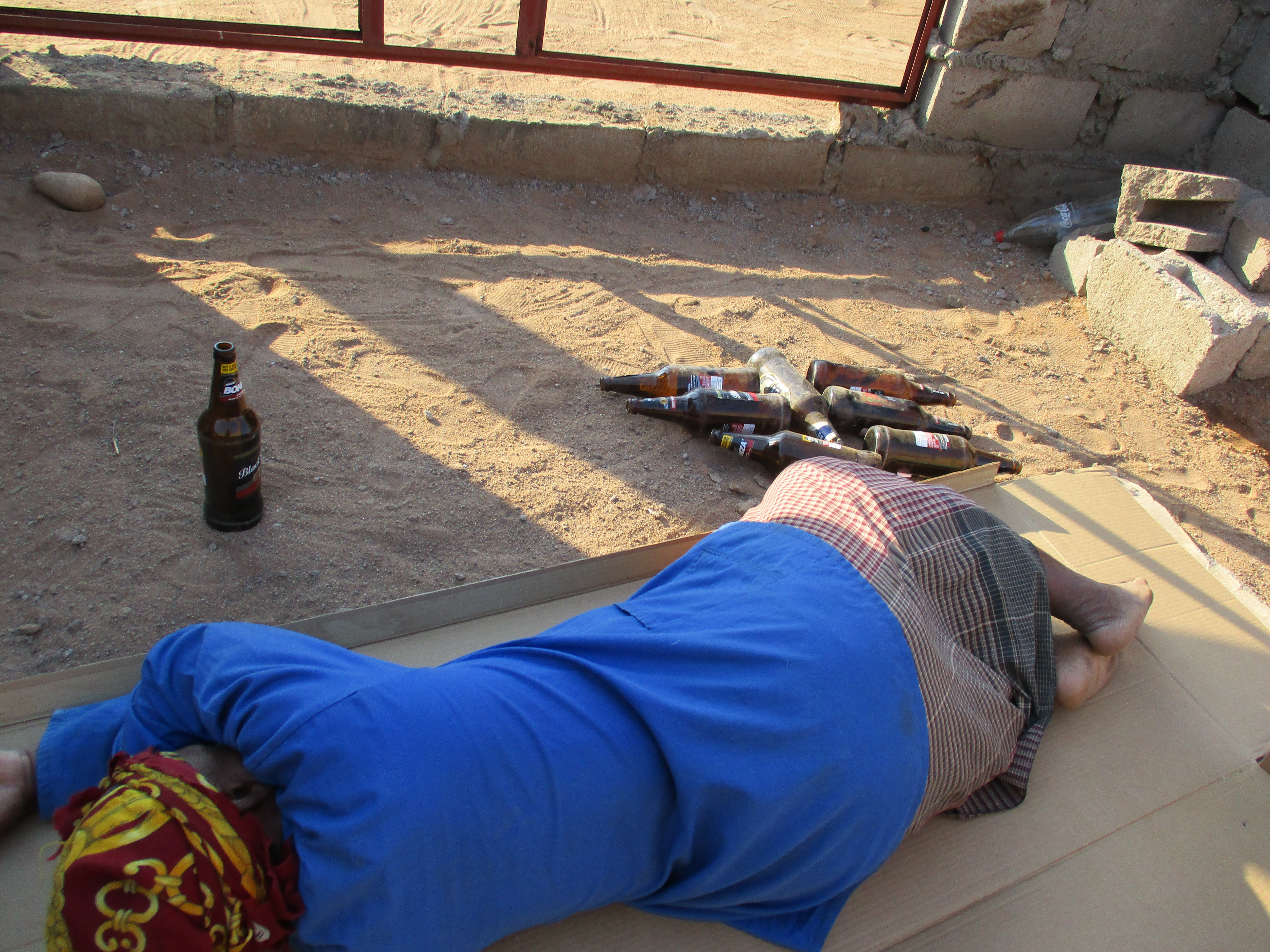

Supplement: Supplemental Material [file ZGHA_A_1726722_SM2442.zip › Supplementary material_06_Older people.JPG]

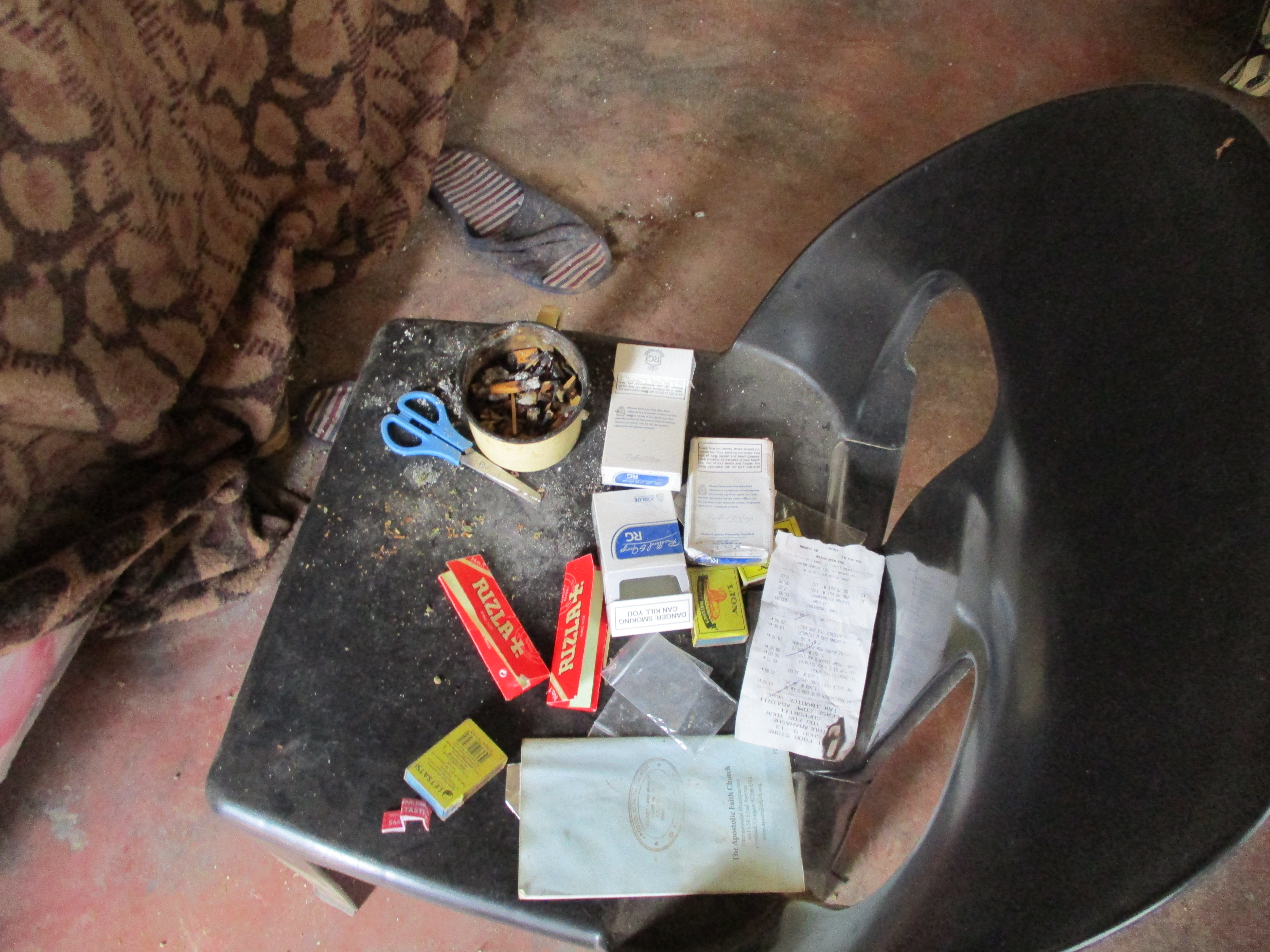

Supplement: Supplemental Material [file ZGHA_A_1726722_SM2442.zip › Supplementary material_07_Drugs at home.JPG]

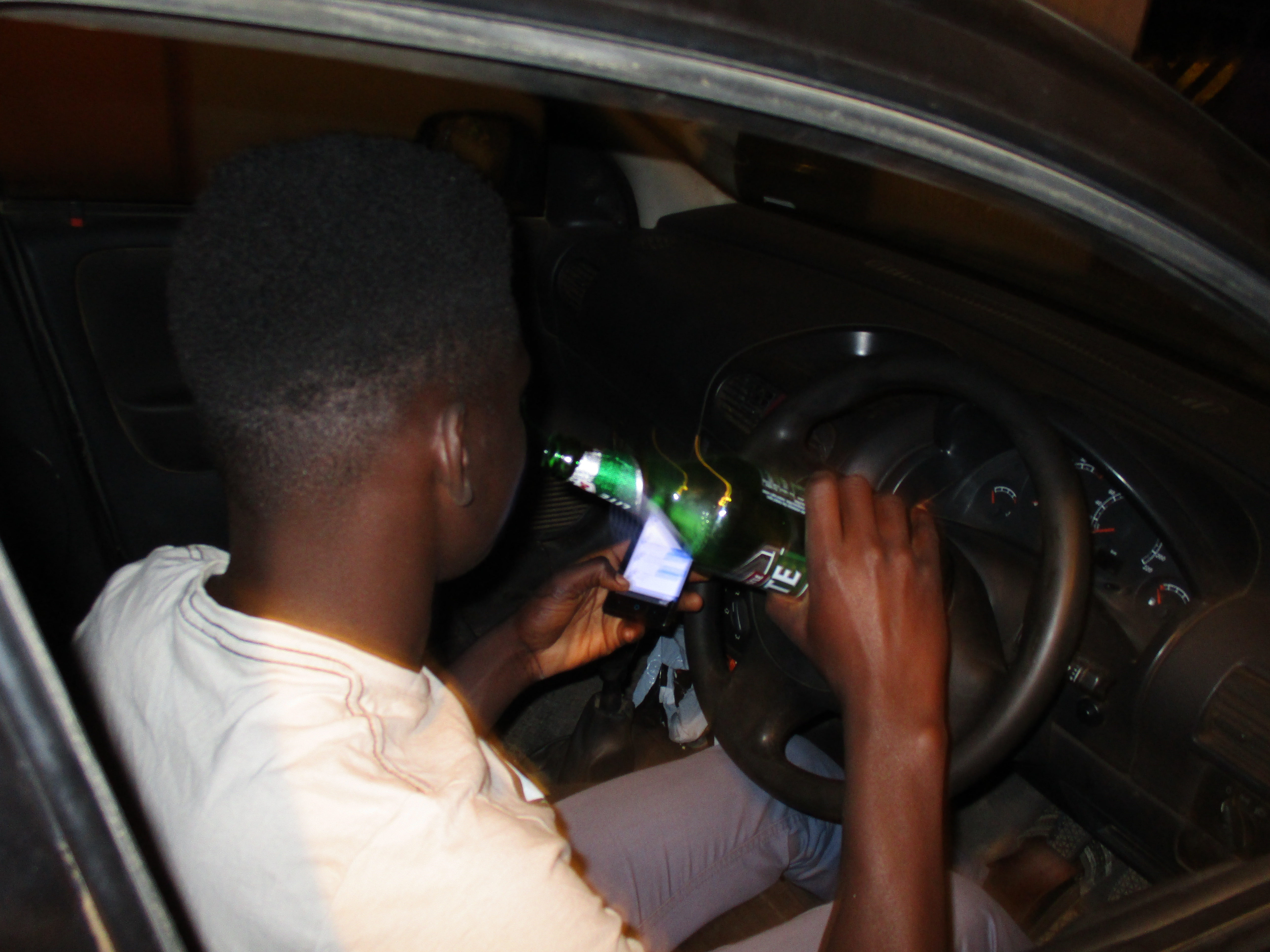

Supplement: Supplemental Material [file ZGHA_A_1726722_SM2442.zip › Supplementary material_08_Drinking driving.JPG]

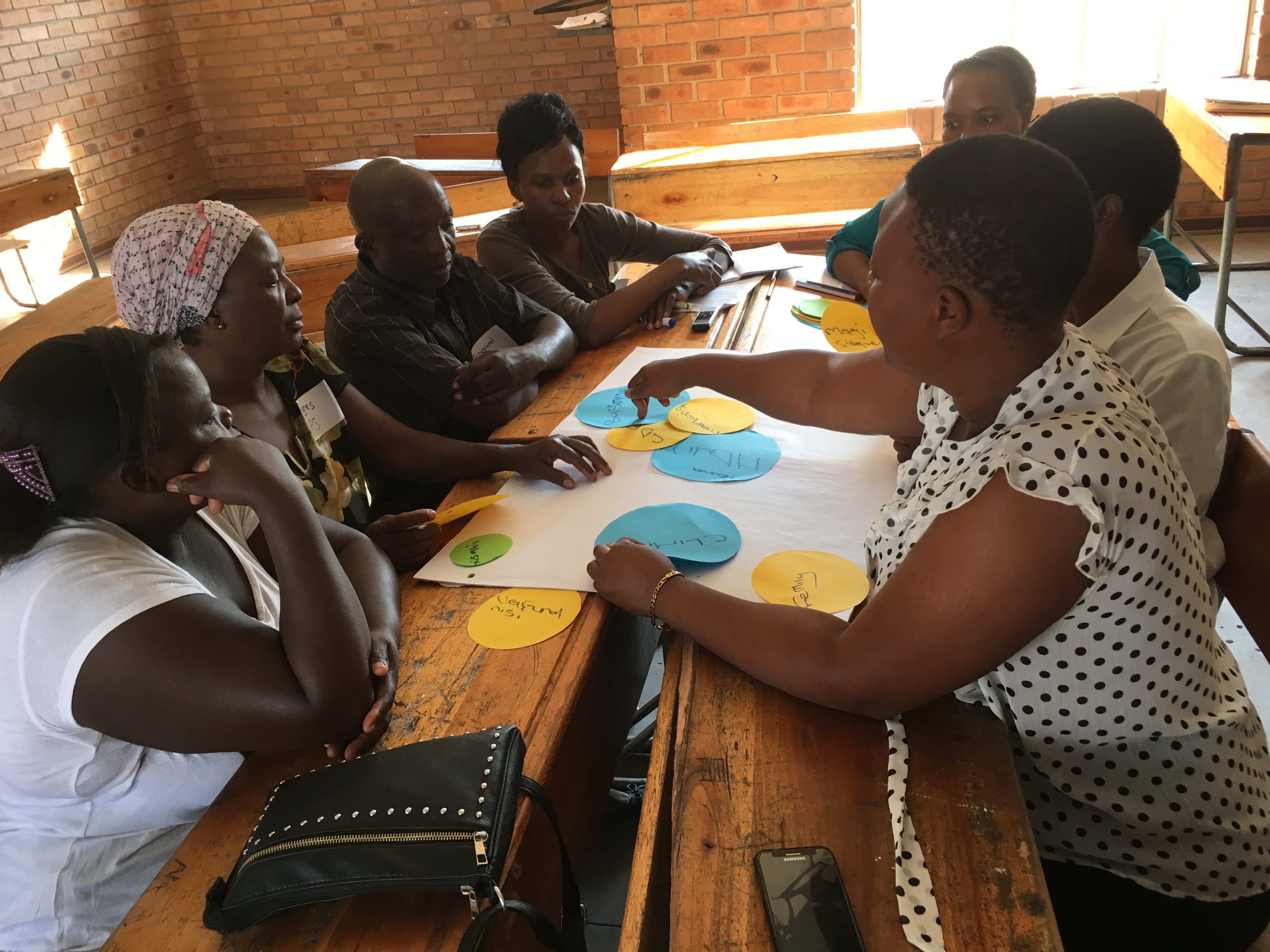

Supplement: Supplemental Material [file ZGHA_A_1726722_SM2442.zip › Supplementary material_09_Planning.jpg]

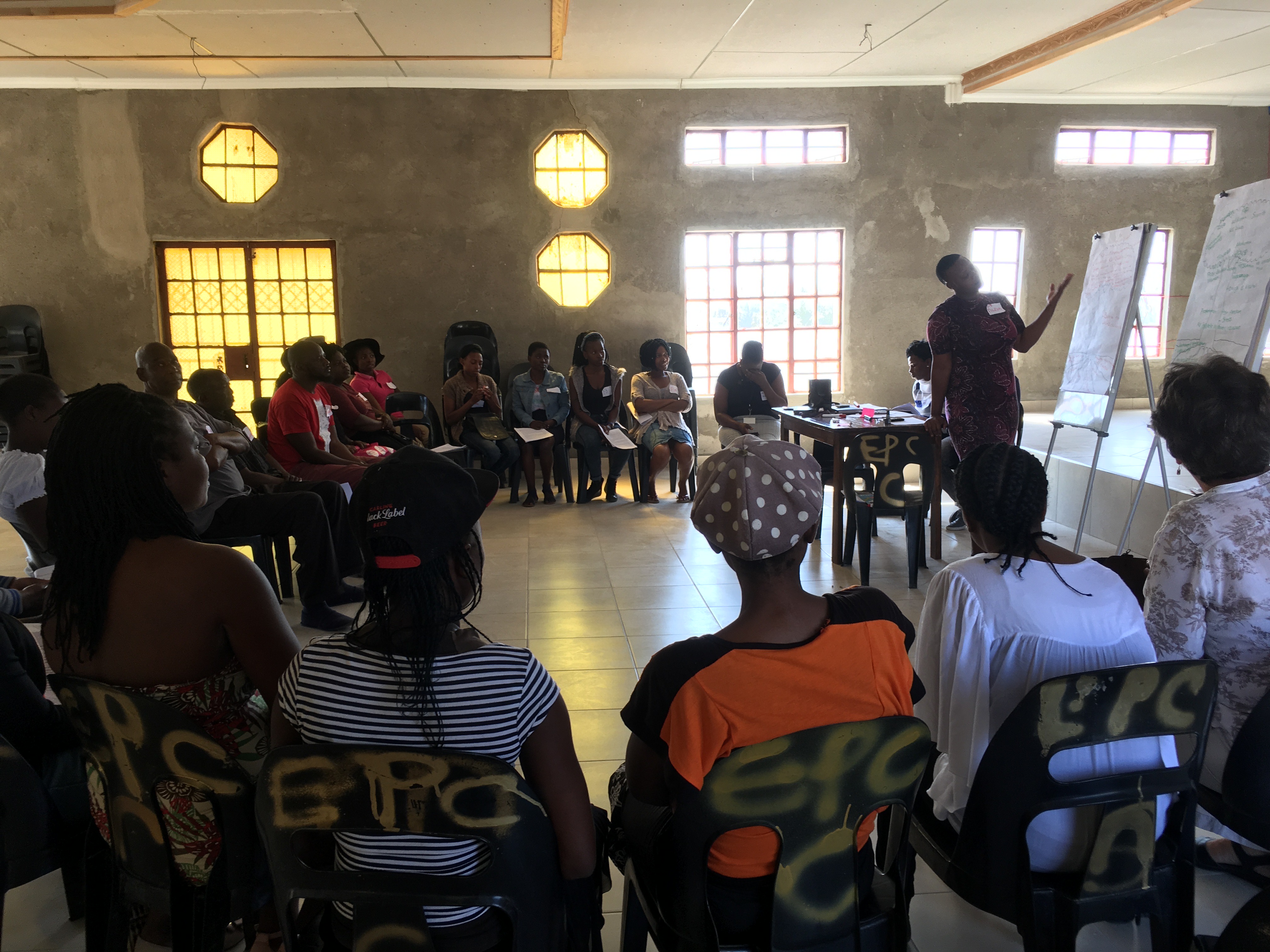

Supplement: Supplemental Material [file ZGHA_A_1726722_SM2442.zip › Supplementary material_10_Collective accounts.jpg]

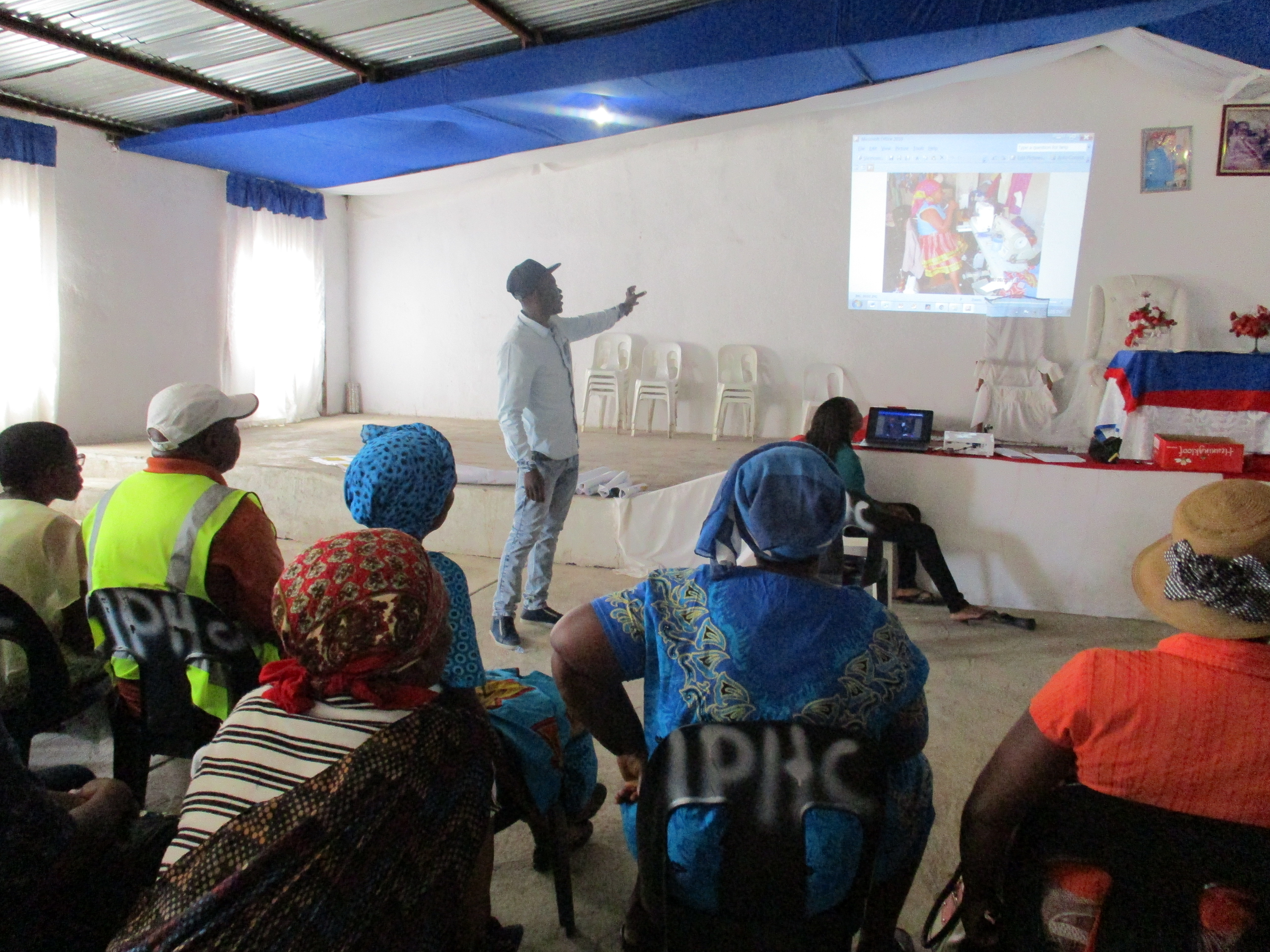

Supplement: Supplemental Material [file ZGHA_A_1726722_SM2442.zip › Supplementary material_11_Visual evidence.JPG]
